# Revolution of Current Dental Zirconia: A Comprehensive Review

**DOI:** 10.3390/molecules27051699

**Published:** 2022-03-04

**Authors:** Ahmed Yaseen Alqutaibi, Omar Ghulam, Majid Krsoum, Suhail Binmahmoud, Hasan Taher, Wael Elmalky, Muhammad Sohail Zafar

**Affiliations:** 1 Department of Prosthodontics, College of Dentistry, Taibah University, Al Madinah 41311, Saudi Arabia; aqaid@taibahu.edu.sa; 2 Department of Prosthodontics, College of Dentistry, Ibb University, Ibb 70270, Yemen; 3 Dental Department, Prince Mohammad bin Abdulaziz Hospital (Ministry of National Guard-Health Affairs), Madinah 42324, Saudi Arabia; omarghlam@gmail.com (O.G.); majid.anas.k@gmail.com (M.K.); hasan-ahmad38@hotmail.com (H.T.); 4 Dental Department, Prince Sultan Armed Forces Hospital, Madinah 42375, Saudi Arabia; subinmahmoud@gmail.com; 5 Department of Restorative Dentistry, College of Dentistry, Taibah University, Al Madinah 41311, Saudi Arabia; wmalky@taibahu.edu.sa; 6 Department of Dental Materials, Islamic International Dental College, Riphah International University, Islamabad 44000, Pakistan

**Keywords:** translucent, monolithic, zirconia, aging, cementation, transformation toughening

## Abstract

The aim of this article is to comprehensively review the revolution of dental zirconia (Zir), including its types, properties, applications, and cementation procedures. A comprehensive search of PubMed and Embase was conducted. The search was limited to manuscripts published in English. The final search was conducted in October 2021. Newly developed monolithic Zir ceramics have substantially enhanced esthetics and translucency. However, this material must be further studied in vitro and in vivo to determine its long-term ability to maintain its exceptional properties. According to the literature, monolithic translucent Zir has had promising results and a high survival rate. Thus, the utilization of this material is indicated when strength and esthetics are needed. Both the materials and methods used for cementation of monolithic Zir have significantly improved, encouraging dentists to use this material, especially when a conservative approach is required. Zir restorations showed promising outcomes, particularly for monolithic Zir crowns supported with implant and fixed dental prostheses.

## 1. Introduction

The use of metal–ceramic restorations has been declining in favor of ceramic prostheses, mainly for esthetic and biocompatibility reasons [1]. On the other hand, ceramics are fragile and brittle in nature, primarily when used as a veneer. Several techniques have been developed to resolve ceramic veneer problems that involve an increase in the zirconia (Zir) translucency and color, enabling the use of the material without needing a veneer. Compared to other ceramic materials, the use of monolithic Zir often dramatically reduces mechanical complications and the need for the preparation of much of the tooth structure and thereby leads to a prosthetic restoration that retains as much of the structure as possible. Monolithic Zir is used for single crowns and has a high survival rate. Currently, the material is being tested to manufacture tooth- or implant-supported fixed partial dentures [2,3,4,5,6,7,8,9]. Dental Zir has traditionally been manufactured mainly from tetragonal Zir crystals with a minor proportion of yttria stabilizer (3Y-TZP); this type is extremely strong but has low translucency [10]. This was accomplished by producing partially stabilized Zir with a greater yttria concentration, such as 4 mol% (4Y-PSZ) or 5 mol% (5Y-PSZ). The c-phase reduces the stress-induced toughening of Zir, resulting in reduced strength and toughness. As a result, the most translucent 5Y-PSZ materials in the anterior zone are limited to single-unit crowns and short-span fixed dental prostheses (FDPs). However, high-stress conditions require stronger restorations, such as multiunit posterior restoration and rehabilitating bruxism patients. Consequently, it is essential to enhance the strength of these ultra-translucent materials. Compared to traditional multilayer restorations, monolithic lithium disilicate offers several benefits [11]. Yttrium-tetragonal Zir polycrystalline (Y-TZP) material can be used in such a case [12,13]. However, the translucency of ordinary Y-TZP is only around 70% of that of lithium disilicate [14].

The ineffectiveness of the traditional etching/silane treatment for Zir has been established [15]. However, surface adjustments are needed; the intaglio surface of Zir is traditionally modified by air abrasion for mechanical retention [16]. Zirconia-based restorations are characterized by good mechanical properties, durability, and tissue biocompatibility, but zirconia is opaque, which limits its use for the posterior area, and its high hardness can abrade the opposing natural teeth. Moreover, due to the crystalline nature of zirconia, etching of the internal surface of zirconia-based restorations with hydrofluoric acid and using adhesive resin cement are not applicable. Hence, zirconia can be cemented using conventional cement, such as glass-ionomer-based cement. Meanwhile, zirconia-reinforced lithium silicate ceramic (ZLS) has better mechanical and optical properties and can be polished intraorally. ZLS can be cemented using adhesive resin cement, which is a great advantage, as it enhances the fracture strength of the restorations [17,18]. This review aims to update the current knowledge about the types, properties, dental applications, and cementation procedures of monolithic Zir.

## 2. Search Strategy

A comprehensive search of the dental zirconia literature was conducted using the PubMed and Embase databases. The final search was conducted in October 2021. Furthermore, journal websites specific to the field of dental materials, prosthodontics, and restorative dentistry were also searched. A manual search was performed by searching the articles and their references. Studies were selected if they were published in English and included data regarding the types, properties, applications, aging, cementation, and transformation toughening of dental zirconia.

## 3. Historical Background

Zirconium is a soft, silver-colored metal extracted as a silicate mineral called zircon. In 1824, Berzelius isolated the metal in an impure state for the first time [19]. Since ancient times, zircon has been regarded as a precious stone [4]. Zirconium dioxide is a crystalline form of zirconium that was first utilized in medicine for orthopedic purposes in 1969. It was recommended as a novel material for hip-head replacement instead of titanium or alumina prosthesis [20].

Because of enhanced awareness about the esthetics, toxicity, and allergic issues associated with specific alloys, both patients and dentists have focused their attention on metal-free tooth-colored restorations. As a result, in the latter half of the 20th century, the development of novel high-strength dental ceramics that appear to be less brittle, less restricted in their tensile strength, and less susceptible to time-dependent stress failure became more prevalent. These features are particularly appealing in prosthetic dentistry, where strength and esthetics are critical [21,22]. In the late 1990s, the first CAD/CAM-fabricated Zir coping was launched to offer a strong and esthetic framework for porcelain-fused-to-Zir (PFZ) restorations. The first popular product was Nobel Procera^®^ Zir (Nobel Bio Care, USA followed by Lava TM Zir (3M ESPE, St. Paul, MN, USA) in the early 2000s [23,24].

## 4. Types of Dental Zir

Dental Zir can be divided into three primary groups based on yttria concentration (Table 1). The first is the hardest, with 3 mol.% Y-TZP (mostly tetragonal). The second group, which contains 4 mol.% Y-TZP, is more transparent, while the third group has 5 mol.% Y-TZP and decreased mechanical characteristics. Mole percentages are responsible for the difference in mechanical and physical properties in each class of Zir. The strongest is opaque Zir containing about 3 mol.% yttria (85–90% tetragonal phase); transparent Zir, with about 5 mol.% yttria, has around 50% cubic phase [25]. Yttria reduces the coefficient of thermal expansion and increases the size of Zir grains. To partially stabilize the tetragonal phase, opaque Zir has 3 mol.% yttria [10]. This composition exhibited the maximum fracture toughness (3.5–4.5 MPam^1/2^) and flexural strength (1200–1500 MPa).

Despite its high strength, the opacity of 3Y-TZP limited its application to posterior teeth; hence, the alumina concentration was decreased from 0.25% by weight to 0.05% to maintain good mechanical properties and translucency. More recent formulations of both 4 and 5 mol.% Y-TZP preserved the alumina at 0.05% by weight while increasing the yttria from 3 to 4 or 5 mol.% [10].

## 5. Fabrication of Zir

The InCeram Zir system (Vita Zanhfabrick, Bad Säckingen, Germany) was the first to use Zir in prosthetic dentistry. The presence of Zir in this ceramic system resulted in a 30–40% improvement in structural strength. Later, CAD/CAM-based industrial ceramic systems improved machining processes for pre-sintered ceramic blocks to produce infrastructures and prosthetic abutments [26]. Prosthetic restorations containing 3Y-TZP can be fabricated by milling pre-sintered blocks and then sintering them at a high temperature or by fully machining sintered blocks. The pre-sintered blocks are machined using CAD/CAM techniques. To counteract sintering shrinkage, the prosthetic restorations are sculptured to be at least 25% larger than the intended design (depending on the batch type). The optimum sintering temperature range is 1350–1550 °C [27].

## 6. Ceramic Infrastructure for Dental Restorations

### 6.1. The Technique of Ceramic Infiltration (Slip-Casting)

InCeram was created using the slip-casting technique as a high-strength infrastructure. The fabrication involves a phase of slip casting of an alumina framework and the addition of a layer of colored glass that is manually infiltrated into this structure, resulting in enhanced resistance (650 MPa) with the potential to accept the ceramic coating. Three infiltrating systems were developed: strengthening with alumina, zirconia, and magnesium. The InCeram Zir system comprises a ceramic body of 67% Al_2_O_3_ and 33% zirconium oxide (tetragonal type). This combination improves the material’s toughness and flexural strength (750 MPa). The inclusion of excessively white Zir gives the combination an opacity equivalent to that of metal–ceramic crowns, reducing translucency in this system [26,28,29].

### 6.2. Compaction Technique—CAD/CAM

Andersson and Oden initially introduced this ceramic system in 1993 under the name of All-Ceram Procera [30]. All-ceramic crowns using this material type are made of non-porous highly sintered pure (99.5%) Al_2_O_3_ infrastructure. The uniaxial vacuum technique involves crushing the powder into a refractory die, which supports a sintering temperature of 1550 °C for 60 min, resulting in a dense crystalline network with a particle size of 4 μm and flexural strength of 600 MPa. The network can also be made from Al_2_O_3_ and Zir using the same compression technique [26].

Most clinical procedures include taking impressions of prepared areas and creating stone die models. A specially designed rotating platform is used to mount the die for scanning purposes. The die is mapped at 360° using a probe with a spherical sapphire tip to record the preparation’s three-dimensional shape with 25,000–50,000 points. The operator works on the image created by the scanner, beginning with the identification of the cervical area, followed by the emerging profile and the thickness of the future infrastructure. The digital design is finished and submitted to a production station via modem. Using this knowledge, an architecture can be built by using a compaction technique called uniaxial or isostatic that involves pressing the powder into a mold to produce a “compressed green” material with the proper shape and sufficient strength. The compression should be performed with care to reduce the density differential due to the features of the process and the conditions of powder agglomeration. The infrastructure is then sintered at high temperatures in a vacuum furnace [31]. Monolithic Zir restorations, which are only fabricated using CAD/CAM technology, have many benefits, such as better flexural strength, the possibility of conservative tooth preparation, decreased antagonist wear, better esthetics, reduced laboratory time, fewer clinical sessions, and a lower likelihood of chipping [32,33,34].

### 6.3. Machining Technique—CAD/CAM

To overcome the drawbacks of casting systems, pre-sintered blocks have been introduced to the market [35]. Moreover, advanced isostatic methods reduce the concentration of errors inside the block during industrial processing, lowering the number of catastrophic failures [27]. The technique starts with the rotation of a CAM ceramic block for 15–50 min using the acquired digital design (CAD), which is generally obtained using a scanner. The resultant piece is neat, but its outline should be double-checked and adapted if necessary before sintering. Items generated after being exposed to machining have a detrimental impact on the process. Pre-sintered block systems have been utilized because they appear less influenced by machining and are more porous. Alternatively, fully sintered blocks are harder, requiring strong equipment, force, and therefore more compressive tension on the block’s outer surface, allowing the block to transition from the tetragonal to monoclinic phase (t→m). Sintering enhances the hardness and fracture toughness of pre-sintered machined infrastructure. However, repeated heat treatments, including the application of feldspathic or glass porcelain to components, appear to negatively impact the material’s fracture resistance [36].

## 7. Properties of Zirconia

### 7.1. Strength of Monolithic Zir

Crowns or single-unit restorations made of monolithic translucent Zir have been observed to have greater overall strength than core material crowns layered with traditional porcelain [37,38]. It has been reported that the material’s flexural strength is two-thirds higher than that of lithium disilicate [39,40]. In addition, translucent Zir has superior fracture resistance to that of lithium disilicate and porcelain-veneered restorations [40]. As previously mentioned, increasing the cubic phase to improve translucency impairs material strength. Consequently, 5Y-TZP should not be used in posterior restorations for more than three units [39]. Due to the lack of transformation toughening, fully stabilized cubic Zir offers fewer mechanical benefits and is suggested for less stress-bearing applications [41]. In 2015, Matsuzaki F et al. compared the flexure strength of three different Zir crowns, namely, Zpex (Tosoh, Tokyo, Japan), conventional opaque TZP, and veneering porcelain (CERABIEN ZIR, Noritake, Tokyo, Japan), and reported that all monolithic TZP with a flexure strength of 1000 MPa was clinically useful for colored translucent TZ [42].

### 7.2. Transformation Toughening

Pure Zir has three main phases: monoclinic (m) at room temperature, tetragonal (t) at 1170 °C, and cubic (c) over 2370 °C. While the (m) phase does not have remarkable mechanical characteristics, the measured incorporation of dopants into the starting powder improves strength and fracture toughness by partially stabilizing the (t) phase within the microstructure at room temperature. Externally induced pressures initiate reversion t → m transformations, which cause individual grains to expand and change shape, absorbing energy and providing damage resistance. Yttria (Y_2_O_3_) has been shown to be the most successful of the various dopants utilized in providing a combination of high strength and toughness. Consequently, 3Y-TZP with a concentration of 3 mol.% (5.2 wt.%) has become the standard dental ceramic for prosthetic restorations [2,43]. The most recent generation of Zir materials has a much higher degree of translucency, resulting in substantially better esthetics. Multilayer high-translucency Zir materials offer a wide range of cosmetic options, particularly for anterior teeth [44].

### 7.3. Aging of Zir

Low-temperature degradation (LTD), often known as aging, occurs in Zir ceramics [45]. The mechanical characteristics of Y-TZP, particularly its strength, are degraded by the spontaneous and gradual transformation of the tetragonal to monoclinic phase. Specific processing conditions, such as humidity, stress, and temperatures of about 200–300 °C, may speed up the process^.^ [45,46,47]. Moreover, according to Chevalier in 2006, this can also happen with water at room temperature [48]. In several experiments, water molecules have been proven to infiltrate the Zir structure when exposed to a hygroscopic environment. The occurrence of multiple oxygen vacancies in the Zir lattice is facilitated by the triatomic property of yttrium, which leads to water molecules in its structure [45]. The diffusion of water produces a lattice contraction, which causes tensile stresses to accumulate on the surface of the Zir grains, causing the tetragonal phase to transform to the monoclinic phase. Surface uplifts and grain pull-out are caused by the increase in volume that occurs when a tetragonal grain becomes monoclinic. This results in microcracks, which allow more water molecules to enter the interior grains. Thus, the initial tetragonal-to-monoclinic transformation continues, traveling deeper and deeper into the bulk of the material. The material breaks as the microcracks expand, and the process continues. This phenomenon may be influenced by microstructural variables such as grain size, stabilizer proportion, residual tensions, and manufacturing defects [47,49]. LTD can be reduced by using smaller grains with uniform yttria distribution and judicious use of oxide sintering additives (e.g., Al_2_O_3_ and TiO_2_) and colorants (e.g., Fe_2_O_3_ and Er_2_O_3_) in low quantities (0.5 wt.%) [47,50]. These additives do not form solid solutions with ZO_2_, and too much of them might induce internal stresses at grain boundaries [51]. Because of the cubic phase, Zir does not undergo a transformation, so Zir with a greater cubic concentration is less vulnerable to aging [25]. Surface roughening of the restoration can occur due to LTD [52], the formation of microcracks [53], and a decrease in the material’s overall mechanical characteristics (20–40% loss in fracture resistance) [54]. The resistance of Zir to LTD is determined by factors such as yttria content [55], grain size [56], cubic phase content [57], Al_2_O_3_ and SiO_3_ content [58,59], and residual stress [49]. To resist aging, the quantity of Al_2_O_3_ present should be more than 0.15 wt.% (in the range of 0.15–0.25 wt.%) [57]. Reducing the Al_2_O_3_ content of Lava Plus (Lava Plus, 3M/ESPES St. Paul, MN, USA) to 0.14 wt.% to help enhance translucency may increase the ability of the material to develop LTD [60]. The solid core of veneered Zir restorations is protected from direct contact with the oral environment by the ceramic veneer. Monolithic translucent Zir, on the other hand, lacks such a barrier and is more vulnerable to LTD [61]. Translucent Y-TZP is significantly more sensitive to LTD than conventional Zir [62]. The flexural strength of several brands of translucent Zir following accelerated aging was investigated recently, and the investigators found that a decrease in the material strength was associated with the depth of LTD [63].

### 7.4. Coloring Effect

Coloring monolithic Zir restorations can be accomplished by either utilizing pre-colored blocks or immersing or brushing white Zir restorations in coloring solutions. Metal oxide solutions such as ferric chloride, manganese chloride, cerium acetate, cerium chloride, bismuth chloride, terbium (III) chloride, chromium (III) chloride, manganese (II) sulfate, and others are used to make coloring liquids. Pre-coloring includes integrating metal oxides such as Fe_2_O_3_ [64], Bi_2_O_3_ [64], CeO_2_ [65], Er_2_O_3_ [65], MnO_2_ [66], and Pr_6_O_11_ [67] into the production of colored nanopowder. The mechanical characteristics of pre-colored Zir may be affected by doping oxides [67]. This occurs due to the crystal lattice alterations caused by exchanging a Zir ion with a metal ion. Kao et al. created powders with various red and yellow combinations [68]. Although powder densification and sintering were enhanced, the size of Zir grains and monoclinic and Fe contents increased, requiring a reduction in the total quantity of the applied oxide [68]. Many investigations have shown that immersing a component in a coloring liquid can reduce its flexural and fracture strength, hardness, and densification [69]. Surface staining or a glaze spray can also alter monolithic translucent Zir. Coloring pre-sintered Zir with an infiltration technique has produced more esthetic results [70]. A finishing and polishing technique, rather than glazing, has also been recommended to achieve a more natural texture [71] because the glazing layer usually wears away after six months [72]. The translucency of partially stabilized translucent Zir was not affected by coloring liquid, whereas the translucency of fully stabilized translucent Zir was reduced. The coloring of partially stabilized translucent Zir lowered its flexural strength. On the other hand, the flexural strength of fully stabilized translucent Zir improved with color [63].

### 7.5. Wear Properties of Zir

The abrasion of material and the wear of opposing teeth are influenced by its surface roughness [73]. The wear characteristics of monolithic Zir were assessed by several in vitro studies. Enamel wear is primarily influenced by the ceramic material’s surface microstructure, the roughness at the point of contact with the antagonist, and other environmental variables [74]. Abrasion of enamel is mainly associated with the ceramic’s hardness and strength but to a lesser extent. In vitro aging of glazed monolithic Zir may change the material’s surface and enhance its roughness [75]. The gloss of Zir was somewhat improved, and the roughness was minimized after toothbrushing [76]. The rest of the restorative materials had the opposite effect [77]. Grinding enhanced surface roughness, while polishing considerably decreased it; nonetheless, friction had little impact on surface roughness and did not affect phase transformation [78].

Polished monolithic Zir restorations show decreased wear of their own surface and that of antagonists [79]. On the contrary, another study found that glazed polished monolithic Zir is more likely to produce wear on the opposing enamel than unglazed polished monolithic Zir [80]. Abrasiveness against steatite was reduced compared to glass-ceramics when the wear was induced by monolithic Zir on different materials [81]. Monolithic translucent Zir with a homogeneous crystal size distribution and orientation resulted in a smooth surface after polishing and less abrasive behavior [82]. Normal enamel wear is 29–38 μm in natural molars and 15–18 μm in natural premolars [83]. In a clinical study, monolithic translucent Zir (Zenostar Zir Translucent) crowns produced more occlusal wear than natural enamel but generated less wear in enamel than other ceramic restorative materials [84,85]. Polished monolithic translucent Zir showed a lower wear rate on the opposing enamel [83].

### 7.6. Optical Properties

The uneven borders of Y-TZP Zir restrict light transmission (anisotropic), whereas cubic-containing Zir is isotropic, which enhances light transmission. Smaller grains allow more light to pass through [86,87]. Light reflection increases when incident light transmission decreases due to the varying indices of refraction of the components that make up 3Y-TZP Zir (alumina, yttria, and colorants). Furthermore, light scattering and absorption are caused by differing indices of the refraction of enamel or dentin and pores in Zir, resulting in an opaque material, which is critical for light-curing Zir restorations. Light transmission is reduced by 50% with a 0.5 mm thick Zir layer and 75% with a 1 mm thick layer. Light transmission is decreased by 85% with moderate staining and a thickness of 0.5 mm and by 95% with a thickness of 1 mm [88]. There are four strategies to overcome these issues. First, the grain size can be increased. The contrast ratio was shown to rise as the Zir grain size was increased [87,88,89,90]. In terms of the diffuse transmission mechanism, less scattering occurs because less light interacts with the grain boundaries [91]. However, the strength of ceramics is reduced due to this drastic shift in the structure [92,93]. Second, a more successful method for achieving more translucent Zir is to reduce the grain size [60,94]. High in-line transmission results in improved translucency [91]. A grain size of 82 nm resulted in translucency with a dental feldspathic porcelain thickness of around 1.3 mm. To create a more translucent material, 77 nm and 70 nm grain sizes are recommended for 1.5 mm and 2 mm thick ceramics, respectively. The material strength is a problematic issue when it comes to grain size. The strength of Zir material is affected by both increasing and decreasing grain size [95]. Third, the yttria dopant content can be increased, which results in a higher cubic phase content of yttria (3% mol yttria) and 90% or more tetragonal Zir, which make up the typical 3Y-TZP [96]. The cubic phase and translucency increase as the yttria concentration rises. The LTD effect may be reduced as the tetragonal phase decreases [97]. Fourth, impurities must be reduced to increase Zir translucency. Ceramics have nonhomogeneous optical properties. Translucency in Zir specimens will be considerably reduced if impurities are less than 0.05% with grain sizes ranging from 200 to 400 nm [86]. The size and quantity of the impurity content have a 50% impact on Zir translucency [98]. As a result, the development of Zir ceramics with lower alumina content has resulted in more transparent Zir (BruxZir (Glidewell) and LavaTM Plus (3M ESPE)); nevertheless, this decrease may accelerate the LTD phenomenon [86]. Increasing the amount of lanthanum oxide to 0.2% mol is another excellent approach for improving translucency [25].

Regarding translucent Zir, the literature proves that the gloss and translucency of Zir vary depending on the brand [99]. An inverse relationship between Zir translucency and strength was observed in in vitro research [100]. The translucency parameter (TP) values in 1 mm thick human dentin and enamel have been reported to be 16.4 and 18.7, respectively [101]. TP ranges from 11.2 to 15.33 for various types of translucent Zir, which is lower than the observed value for lithium disilicate (16.89) [39]. Despite all of the attempts made over the previous decade to increase the translucency of dental Zir, it is widely acknowledged that translucent Zir beyond 0.5 mm in thickness remains mainly opaque and is still less translucent than ordinary lithium disilicate [102]. Reflection spectrometry and MARC calibration of five different materials, namely, Prettau (PRT, ZIRKONZAHN), Bruxir (BRX, Glidewell), Zenostar (ZEN, Wieland), Katana (KAT, Noritake), and Prettau anterior (PRTA, Zirconzahn), showed that translucency is brand- and thickness-dependent. Fully stabilized Zir is more translucent than partially stabilized Zir [103]. However, in 2017, Kwon et al. measured the translucency of different materials by spectrometry and concluded that the translucency parameter of Katana HT was significantly lower than that of Katana UTML, which was remarkably lower compared to e.max CAD LT [39].

### 7.7. Survival of Zir Restorations

Zir restorations have similar survival rates to other types of dental restorations; for instance, single crowns with a Zir core had a five-year survival rate of 91.2% [104], and success rates for porcelain-veneered Zir FDPs varied from 67% to 100% in a follow-up period of 5–10 years [105,106,107,108]. At the same time, a recent systematic review found that the survival rate of Zir FDPs was 89.43%, with chipping of the veneering ceramic occurring in 16.97% of cases [109]. Furthermore, it was reported that the Zir framework FDP had a 100% 5-year survival rate, which is considered statistically greater than that of metal–ceramic resin-bonded FDPs [110].

In addition, it has been found that an anterior Zir prosthesis provides the best clinical results. The most prevalent problems with these restorations included debonding (15%) and chipping (4.1%). Regarding inlay-retained FDPs, a success rate of 95.8% during a mean follow-up time of 64.4 months was reported [111]. The survival rate was 86.7% for crowns and 92.3% for FDPs, whereas 100% survival of 3–12-unit FDPs was reported during a follow-up period of 3–7 years [112]. Data from two commercial dental laboratories were used to study the failure rate of monolithic Zir restorations owing to fracture. A total of 3731 anterior restorations were included, as well as 36,096 posterior restorations. The total fracture rate was relatively low at 1.09% over a period of five years; fracture rates for anterior restorations were 2.06% and 0.99% for posterior restorations, and fracture rates for anterior crowns were 0.97% and 0.69%, respectively [113].

Fractures occurred in 3.26% of anterior FDPs and 2.42% of posterior FDPs, indicating that anterior restorations of FDPs have a slightly higher fracture rate. In addition, single monolithic Zir resin-bonded bridges had a survival rate of 82.7% after 36.2 months of monitoring. Debonding was the most common problem reported in this research [114]. Survival rates for implant-retained monolithic crowns varied from 97.1% to 100% for two years [115,116,117] and 98.4% for up to three years of follow-up [118]. The reported survival rates of FDPs include 91.7% after two years [116], 100% after three years [118], and 97.4% after five years [119]. Additionally, the survival rate of full-arch fixed prostheses varied from 88% to 100% [120,121,122,123]. There were significant differences in the methodology, sample size, and commercial items utilized among these studies; hence, no definite conclusions can be drawn, but the results are encouraging.

## 8. Applications in Dentistry

### 8.1. Zir-Based Dental Posts

Metal posts can cause adverse esthetic outcomes in circumstances when all-ceramic restorations are utilized to restore anterior teeth, including the staining of translucent all-ceramic crowns [124]. Corrosive responses with prefabricated posts can also create problems in oral environments, such as a metallic taste, mouth burning, sensitivity, and discomfort [125]. As a result of these issues, translucent posts made of Zir and other ceramic materials have been developed. Smooth, tapered, and parallel Zir posts are available, as well as those that taper at the apex and are parallel at the coronal aspect. The apical zenith is rounded to reduce stress accumulation at the root apex. Zir posts are highly biocompatible and radiopaque, and they have good light transmission in root and coronal restorations. In a clinical trial, Zir ceramic posts had high success rates [126]. Similarly, Zir posts with direct composite cores had an excellent clinical success rate after 4.7 years [127]. Zir posts have certain advantages in terms of esthetics and biocompatibility [128]. However, there are certain limitations, such as rigidity, lack of ductility, difficulty working with smaller sizes, and the need for retreatment. [129]. After dynamic loading and thermocycling, Zir posts had poor resin-bonding capacities in radicular dentine [130]. Similarly, compared to serrated metal posts, Zir ceramic posts had lower retention values [128]. Recently, it was confirmed that a custom-made Zir post and core could be fabricated with CAD/CAM either directly by scanning the canal space [131] or indirectly after taking an impression and scanning the cast replica [132].

### 8.2. Zir-Based Crown and Bridge

For crowns and bridges, various types of Zir frameworks have been used [133,134]. Zir frameworks provide new prospects for metal-free permanent partial dentures and single-tooth prostheses and have shown promising early clinical outcomes [135]. A study used the DCS President^®^ technology to produce 65 Zir bridges and followed up recipients for an average of three years. The results showed that 6% of the bridges had minor veneering material peeling, indicating an 86% cumulative survival rate [136].

### 8.3. Zir-Based Implant Abutments

According to in vitro studies, the biocompatibility of Zir is improved compared to that of titanium oxide and is equivalent to that of alumina. No cytotoxicity, carcinogenicity, mutagenicity, or chromosomal changes have been found [35]. Zir is used as an implant-supported restoration because of its excellent toughness and lower modulus of elasticity. Zir has some benefits over alumina in stabilized and transformation-toughened forms, which helps to overcome the problem of alumina brittleness and possible implant failure [137]. These abutments stand out because of their tooth-colored appearance, high tissue compatibility, and reduced plaque buildup [138,139,140]. An in vivo investigation demonstrated a high successful cumulative survival rate (98–100%) when using Zir and Al_2_O_3_ abutments [140]. A cumulative survival rate of 100% for 53 Zir was found in a prospective trial with a four-year monitoring period [141]. It was reported that a patient who had multiple implant-supported Zir crowns suffered an incidence of metal sensitivity [142]. Successful osseointegration was achieved in both examples. Despite this, Zir is a highly reliable abutment biomaterial for implant-supported crowns and FDPs. However, veneering porcelain fractures is a barrier to the clinical success of Zir-based implants. Zir fractures are the result of a technological issue [143,144]. It was reported that Zir’s clinical long-term effectiveness in fixed implant prosthodontics is in question due to veneering ceramic fractures and Zir’s sensitivity to aging [145]. Restorations containing 3Y-TZP have been recommended as an alternative to titanium abutments and implants due to their optical properties, higher corrosion and wear resistance, enhanced biocompatibility, and reduced affinity for plaque accumulation and peri-implantitis. However, clinical investigations have shown a greater risk of early fracture in cases of Zir implants compared to titanium implants [146,147]; thus, mechanical integrity becomes the primary issue.

### 8.4. Zir Bar-Retained Implant Overdenture

The bar attachment used to retain overdentures is commonly manufactured from base metal and titanium alloys; however, due to its excellent biocompatibility, strength, and natural color, Zir has emerged as a potential material for constructing bar attachments [148]. Furthermore, a Zir bar can be easily manufactured utilizing CAD/CAM technology, eliminating many of the technical processes and errors associated with traditional casting techniques [149].

### 8.5. Single-Retainer Zir Resin-Bonded Bridge (RBB)

If fundamental criteria are met, the single-retainer ceramic RBB has been demonstrated to be the most predictable of the alternatives. In the past, the most frequently used materials were InCeram (alumina) and e.max (lithium disilicate). Zir currently seems to be the material of choice when it comes to connector strength [150,151].

### 8.6. Zir Esthetic Orthodontic Brackets

Zir has been used to fabricate esthetic orthodontic brackets [152]. Alumina ceramic brackets have been replaced by polycrystalline Zir brackets due to the enhanced toughness [153]. Polycrystalline Zir brackets are less expensive than monocrystalline Al_2_O_3_ ceramic ones; however, it is opaque and less esthetically appealing. Both stainless steel and nickel–titanium archwires have been observed to exhibit good sliding characteristics, as well as decreased plaque adhesion [154].

### 8.7. Veneer

Zir veneers have undergone several modifications in their microstructure and compositions in recent years [42] to enhance translucency without sacrificing mechanical properties [155]. Therefore, translucent Zir is regarded as a suitable material, with indications for crowns, posterior and anterior monolithic FDPs, conventional veneers, and ultra-thin veneers [156]. Ultra-translucent Zir veneers have a minimum thickness of 0.1–0.3 mm, a more conservative option than glass-ceramic restorations [157]. Because polycrystalline Zir is chemically inert and hard to etch using hydrofluoric acid (4–10%), it has a lower adhesion than silica-based ceramics (acid-sensitive) in conditions with limited mechanical retention of the preparation [70]. In vitro studies on veneers have revealed that Zir has a higher resistance to fracture than feldspathic veneers and lithium disilicate, which can be viewed as a significant benefit because the proof and luting phases of ultra-thin veneers are far less critical than conventional glass-ceramics. However, Zir veneers might debond due to a lack of efficient adherence to resin cement [158]. The use of translucent ultra-thin Zir veneers results in pleasing esthetics, although more research is needed to confirm this form of therapy [159].

### 8.8. Inlay-Retained Zir Fixed Dental Prosthesis

Posterior tooth loss can be replaced utilizing various treatment methods and materials. In cases where the dental implant placement is contraindicated, a resin-bonded minimally invasive technique offers an alternative to conventionally fabricated FDPs with minimal tooth preparation [160]. This is also true in the case of previously restored abutment teeth [161]. Existing restorations may reduce the amount of tooth structure removed and help the inlay-retained FDP remain in place longer, making it a more conservative alternative to the full-coverage FDP [162]. In addition, it also enables more of the tooth structure to be preserved [163] and simplifies periodontal evaluation [164]. Patients with good oral hygiene and low caries susceptibility are candidates for inlay-retained Zir FDPs. On the other hand, severe parafunctions, a lack of marginal enamel, significant crown deficiencies, and abutment tooth movement are considered contraindications to this type of restoration [165].

## 9. Cementation of Zir Restorations

In the commercial market, Zir competes with glass-ceramics and silica-based feldspathic porcelains, which are preferred over Zir due to their high glass content, excellent translucency, and natural enamel-like appearance. The capacity of silicate ceramics to be acid-etched and silanized promotes resin bonding and reinforcing [166]. For many years, the clinical performance of luted prostheses has been evaluated by assessing the marginal fit and microleakage [167]. Microleakage is associated with various issues, including the loss of bonding, staining, secondary caries, pulpal inflammation, postoperative sensitivity, and plaque accumulation. The Zir framework’s high mechanical properties may allow for adhesive bonding or traditional cementation [168]. Although Zir restorations are deemed “cementable,” some benefits from the use of composite resin-luting agents can be realized. Resin-bonded fixed prostheses or veneers are examples of Zir restorations, which are thin, have minimal robustness, lack retention, or depend on resin bonding [169]. The effectiveness of resin bonding is dependent on selecting suitable materials and treating the tooth and restorative bonding surfaces properly [166].

Several bonding protocols have been recommended. However, the desired long-lasting bond can be achieved by using an adhesive resin luting material, which integrates specific bonding phosphate monomers, especially 10-methacryloyloxydecyl dihydrogen phosphate (MDP), after intaglio surface pre-treatment with air-particle abrasion [170,171]. Air abrasion results in t→m (monoclinic) transformation and produces a protective surface layer on 3Y-TZPs, therefore creating strength-limiting surface defects [172]. Air abrasion can either diminish [173,174] or enhance [16,175] the flexural strength of 3Y-TZP depending on operating parameters, including the applied air pressure, type, and size of abrading particles. Furthermore, it was discovered that sandblasting translucent Zir with Al_2_O_3_ did not increase surface roughness. Sandblasting affects residual stress and the crystalline phase composition in samples [176]. It appears that the type of cement used has little effect on the stress distribution of single monolithic translucent Zir [177]. A three-step bonding method is recommended to obtain long-lasting and strong bond strengths to Zir. This method includes the use of aluminum oxide to perform air-particle abrasion of the intaglio surface, followed by using a specific Zir primer and, finally, the use of dual-cure or self-cure composite resin cements. This procedure is called the “APC Zir-bonding concept” to make it easier to remember [178].

It was reported that there was stronger bonding between resin material and Zir when using a porcelain coating on the bonding surface. This porcelain coating can be surface-conditioned using hydrofluoric acid etching and a silane coupling agent to improve the bonding strength. According to a recent meta-analysis, resin-based cement provides the best bonding to Zir [179]. For the cementation of ceramic restorations, dual-cure materials are a common choice [180]. In in vitro research comparing the bond strength of 3Y-TZP, 5Y-TZP, and lithium disilicate samples bonded with resin cement, shear bond strength showed no significant difference [39]. Light attenuation and subsequent cement curing also impact light-cured materials for cementing ceramic restorations. The composition of the resin cement, the intensity of the curing light, the curing time, and the distance between the light-guiding tip and the restoration are all elements that influence light polymerization [181]. As a result, light transmission through the restoration is influenced by ceramic composition, shade, thickness, translucency, defect size and distribution, and porosity [182,183,184]. Using a try-in paste cement might be helpful in terms of esthetic needs [185].

It was discovered that the curing time for thin ceramic (0.5 mm) must be extended by 40% compared to the curing time for a resin composite without ceramic (not a cement material). The curing time must be doubled when the thickness is increased to 1 mm [186]. However, saliva contamination during the try-in may impair the interface with the resin cement, which is a practical issue when bonding to Zir restorations [187]. Other cleaning procedures, such as using alcohol or organic solvents, are not very effective [187,188,189]. Particle abrasion effectively eliminates impurities to restore the bonding strength to its previous levels [187,190,191]. However, to eliminate impurities, a relatively new and commercially available product (Ivoclean; Ivoclar Vivadent, Schaan, Liechtenstein) made of hyper-saturated Zir particles was created. Contaminants introduced by the Ivoclean solution can be removed from the repair surface. Initial assessments of the cleaning solution confirmed the effectiveness [188,190,191]. The findings of this investigation led to several clinical recommendations. Before applying an MDP primer, any salivary contamination should be cleansed with Ivoclean or further particle abrasion. The bonding seems to re-establish with 20 s of water washing in clinical circumstances when Zir becomes contaminated when treated with a combination of MDP, such as intraoral bonding or using MDP before trying in [192].

## 10. Future and Challenges of Dental Zir

One of the significant challenges is using tough ceramic as a core to support overlaying porcelain veneers [193]. As previously stated, attention has switched to monolithic Zir to avoid veneer chipping and delamination and to reduce material thickness requirements. The esthetic properties of monolithic Zir are enhanced using various dopants in the starting powders. For example, incorporating 0.2 mol% Al_2_O_3_ into 3Y-TZP improves aging resistance and translucency; however, it degrades mechanical characteristics [25]. Furthermore, the improvement in translucency is less significant compared to 5Y-PSZ. Enhanced strength and aging resistance have been achieved using Zir-toughened Al_2_O_3_, but opacities still prevent applications for anterior restorations [194]. Experimenting with various dopants and sintering methods to enhance translucent phases represents a promising topic for further study, which should include proper consideration for the general trade-off in terms of esthetics and mechanical properties [10]. The scattering of light due to grain boundaries and microstructural imperfections enhances the opacity of the material. Alternatively, reducing the grain size to well under the wavelength of light increases Y-TZP transmittance. According to traditional light-scattering models, a grain size less than 100 nm is required for satisfactory transmittance in Y-TZP ceramics [86].

If starting with well-dispersed homogeneous nanopowders containing regulated quantities of stabilizing additives, the fabrication of Zir with nanoparticles is extremely hard. Zir nanopowders are currently commercially available to minimize porosity and enhance grain development while sintering; new fabrication techniques must be designed. These processing methods are currently being developed [10]. The mechanical characterization of nanostructured 3Y-TZPs shows increased strength, corresponding to reduced inherent defect sizes. In addition, the small grain size may impede the reversal of t→m transformation [195]. The increased translucency eliminates the need for veneering.

## 11. Conclusions

Newly developed multicolor monolithic Zir exhibits superior translucency and esthetic properties. However, further in vitro and in vivo trials are needed to validate their exceptional properties. Monolithic translucent Zir has had promising results and high survival rates. Thus, the use of this material is indicated when strength and esthetics are needed. Cementation of monolithic Zir has shown significant improvements in properties, encouraging dentists to use this material, especially when a conservative approach is required. A few short-term investigations on Zir restorations showed promising outcomes, particularly for implant-supported single crowns and FDPs. Because data are scarce, well-designed clinical trials are needed to shed light on prognosis and long-term survival concerns.

## Figures and Tables

**Table 1:** Various types of dental Zir based on yttria concentration.

3Y-ZP	4Y-TZP	5Y-TZP
IPS e.max^®^ ZirCad LT and MO (Ivoclar Vivadent) ivoclarvivadent.com (accessed on 22 December 2021)	IPS e.max ZirCAD MT (Ivoclar Vivadent) ivoclarvivadent.com (accessed on 22 December 2021)	Cercon^®^ XT, **Dentsply Sirona**, dentsplysirona.com (accessed on 22 December 2021)
BruxZir^®^ (Glidewell Laboratories) glidewelldental.com (accessed on 22 December 2021)		BruxZir Anterior (**Glidewell Laboratories**) glidewelldental.com (accessed on 22 December 2021)
KATANA™ HT (Kuraray Noritake) KATANAZir.com (accessed on 22 December 2021)	KATANA™ ST/STML (Kurary Noritake) KATANAZir.com (accessed on 22 December 2021)	KATANA™ UT/UTML (Kurary Noritake) KATANAZir.com (accessed on 22 December 2021)
Lava™ Plus, 3M ESPE		Lava Esthetic, 3M ESPE
Zpex 3Y (Tosoh)	Zpex^®^ 4, Kraun, (Tosoh) kraun.eu (accessed on 22 December 2021)	Zpex Smile (Tosoh)
Zenostar MO (Wieland Dental)	Zenostar MT (Wieland Dental)	Prettue Zir (Zirconzhan)
